# Classification of Lapses in Smokers Attempting to Stop: A Supervised Machine Learning Approach Using Data From a Popular Smoking Cessation Smartphone App

**DOI:** 10.1093/ntr/ntad051

**Published:** 2023-03-27

**Authors:** Olga Perski, Kezhi Li, Nikolas Pontikos, David Simons, Stephanie P Goldstein, Felix Naughton, Jamie Brown

**Affiliations:** Department of Behavioural Science and Health, University College London, London, UK; SPECTRUM Consortium, London, UK; Institute of Health Informatics, University College London, London, UK; UCL Institute of Ophthalmology, University College London, London, UK; Centre for Emerging, Endemic and Exotic Diseases, Royal Veterinary College, London, UK; Weight Control and Diabetes Research Center, The Miriam Hospital, Providence, RI, USA; Department of Psychiatry and Human Behavior, Alpert Medical School of Brown University, Providence, RI, USA; Behavioural and Implementation Science Research Group, School of Health Sciences, University of East Anglia, Norwich, UK; Department of Behavioural Science and Health, University College London, London, UK; SPECTRUM Consortium, London, UK

## Abstract

**Introduction:**

Smoking lapses after the quit date often lead to full relapse. To inform the development of real time, tailored lapse prevention support, we used observational data from a popular smoking cessation app to develop supervised machine learning algorithms to distinguish lapse from non-lapse reports.

**Aims and Methods:**

We used data from app users with ≥20 unprompted data entries, which included information about craving severity, mood, activity, social context, and lapse incidence. A series of group-level supervised machine learning algorithms (eg, Random Forest, XGBoost) were trained and tested. Their ability to classify lapses for out-of-sample (1) observations and (2) individuals were evaluated. Next, a series of individual-level and hybrid algorithms were trained and tested.

**Results:**

Participants (*N* = 791) provided 37 002 data entries (7.6% lapses). The best-performing group-level algorithm had an area under the receiver operating characteristic curve (AUC) of 0.969 (95% confidence interval [CI] = 0.961 to 0.978). Its ability to classify lapses for out-of-sample individuals ranged from poor to excellent (AUC = 0.482–1.000). Individual-level algorithms could be constructed for 39/791 participants with sufficient data, with a median AUC of 0.938 (range: 0.518–1.000). Hybrid algorithms could be constructed for 184/791 participants and had a median AUC of 0.825 (range: 0.375–1.000).

**Conclusions:**

Using unprompted app data appeared feasible for constructing a high-performing group-level lapse classification algorithm but its performance was variable when applied to unseen individuals. Algorithms trained on each individual’s dataset, in addition to hybrid algorithms trained on the group plus a proportion of each individual’s data, had improved performance but could only be constructed for a minority of participants.

**Implications:**

This study used routinely collected data from a popular smartphone app to train and test a series of supervised machine learning algorithms to distinguish lapse from non-lapse events. Although a high-performing group-level algorithm was developed, it had variable performance when applied to new, unseen individuals. Individual-level and hybrid algorithms had somewhat greater performance but could not be constructed for all participants because of the lack of variability in the outcome measure. Triangulation of results with those from a prompted study design is recommended prior to intervention development, with real-world lapse prediction likely requiring a balance between unprompted and prompted app data.

## Introduction

About 40% of smokers make a quit attempt each year,^1^ but of these, less than 5% who use no support remain abstinent for one year.^2,3^ Smoking lapses (ie, a temporary smoking episode after the quit date) are a key reason why people fail to quit, as they quickly lead to full relapse.^4,5^ The majority of those who experience an initial lapse during a quit attempt progress to full relapse, and this transition takes ~19 days on average.^5^ A recent Cochrane review concluded that brief, skills-based behavioral interventions do not help to prevent relapse.^6^ Studies harnessing real time, ecological momentary assessments (EMAs) of smokers’ internal and external contexts indicate that lapse risk fluctuates over time, with different contextual and psychological variables important for different individuals. For example, situational cravings, stress, negative affect, and the presence of other smokers are associated with momentary lapse incidence.^7–11^ With hardware and software advances, real-time lapse risk support can be delivered via technology-mediated just-in-time adaptive interventions (JITAIs). Here, we used longitudinal, observational, unprompted craving feature data from a popular smoking cessation app (“Smoke Free”) to train and test supervised machine learning algorithms to classify lapse incidence at the group- and individual-level, with a view to informing the development of a JITAI to prevent lapses in smokers attempting to quit.

Supervised machine learning has previously been used to classify or predict smoking behavior in non-treatment-seeking smokers,^12^ smoking environments from images taken in non-treatment-seeking smokers’ daily lives,^13^ smoking “opportunity”^14^ or cravings^15,16^ in smokers attempting to quit, time to first lapse in smokers attempting to quit,^17–19^ and lapse “vulnerability” in smokers attempting to quit.^20^ Although findings to date indicate that supervised machine learning algorithms can predict several smoking-related events with acceptable accuracy and precision, sample sizes tend to be small (*N* = 5 to 349 participants), with few studies focusing specifically on the classification or prediction of lapses in smokers attempting to quit—thereby missing and important opportunity for future intervention. In addition, the few studies that have focused on lapse incidence or risk prediction tend to have deployed specialist equipment, including bespoke wearable physiological sensors,^19,20^ which are not yet widely available in the general population of smokers. That said, machine learning algorithms incorporated into widely used smart watches for detecting smoking events are currently being developed, with a view to extending the work to lapse prediction in smokers attempting to stop.^21^

As a further consideration, previous research has focused on the development of group-level prediction algorithms. However, with growing evidence that lapse risk is idiosyncratic, with different factors important for different individuals,^7–11^ it is important to examine the performance of group-level algorithms for new, “unseen” individuals (ie, people who have just started using a smoking cessation app). Alternatively, algorithms using a combination of data from the group and a proportion of data from each individual—referred to as a “warm start”^22^—may lead to improved accuracy at the individual-level, important for the development of technology-mediated JITAIs. Such a hybrid approach has recently been applied within the weight loss domain, with the best-performing algorithms identified in preliminary development work used to underpin a smartphone-based JITAI to prevent dietary lapses.^23–25^ However, to the best of our knowledge, this approach has not yet been tested in the smoking cessation domain.

To develop algorithms that can be directly implemented in real-world contexts, it is important to study smoking lapses in the context of empirically supported yet popular and widely available smoking cessation tools. The Smoke Free app includes behavior change techniques that research suggests are likely to improve the chances of quitting.^26^ The app is live on app stores and has a large user base with >1 million global downloads per year. The “pro” (paid) version of Smoke Free includes a craving feature, which allows users to self-initiate a new entry when experiencing a craving and asks them to indicate whether they have lapsed, their craving strength, how they are feeling, what they are doing and who they are with. With its large user base and embedded features to assess (near) real-time lapse risk in smokers attempting to stop, the Smoke Free app acts as a useful testbed for the development of supervised machine learning algorithms to distinguish lapse and non-lapse reports.

Specifically, this study aimed to address the following objectives:

1. To develop a series of group-level algorithms (ie, algorithms trained on the entire dataset minus a holdout sample) and evaluate their ability to classify lapses for out-of-sample observations (ie, randomly selected observations in the dataset).
2. To evaluate the ability of the best-performing group-level algorithm to classify lapses for out-of-sample individuals (ie, each individual in the dataset).
3. To develop a series of individual-level algorithms and evaluate their ability to classify lapses for out-of-sample individual observations (ie, randomly selected observations in each individual’s dataset).
4. To evaluate the ability of a hybrid (ie, group- and individual-level) algorithm to classify lapses for out-of-sample individuals.

## Methods

### Study Design

This was a longitudinal, observational study with unprompted (ie, self-initiated) repeated measures of lapse incidence nested within participants. The Smoke Free app is available in commercial app stores (ie, the Apple App Store and the Google Play Store). The study protocol and exploratory analysis plan were preregistered on the Open Science Framework (osf.io/rx3pn). Decisions as to whom to include in the analytic sample (eg, users with ≥10 or ≥20 craving feature entries made within the first 3 months after app registration) were made based on the empirical distribution, following inspection of the data.

### Eligibility Criteria

Smokers were eligible for inclusion if they: (1) had purchased the “pro” version of Smoke Free on or after January 1st, 2020 (when the app had migrated to a new backend server), (2) had their phone set to English language, and (3) had set a quit date for the 16-day period beginning 2 days before (t_−2_) and ending 14 days after (t_+14_) their date of registration (t).

### Sample Recruitment

There was no active recruitment; participants had voluntarily downloaded the Smoke Free app and agreed for their data to be analyzed by researchers at University College London via an in-app agreement. Ethical approval for the study was obtained from UCL’s Research Ethics Committee (Project ID: 15297/003).

## Measures and Procedure

When downloading the Smoke Free app, users are asked to provide information on time to first cigarette (ie, ≤5 minutes, 6–30 minutes, 31–60 minutes, and >60 minutes), cigarettes smoked per day, and set a quit date. No other baseline characteristics are recorded.

### Outcome Variable

The outcome variable was whether participants self-reported a lapse (no vs. yes) via the app’s craving feature (see Supplementary Material, Figure S1). Participants’ interactions with the craving feature were unprompted. Participants were not given any specific instructions as to when or how often to use the craving feature.

### Explanatory Variables

#### Time-Invariant Variables

The time-invariant explanatory variables included time to first cigarette after waking up (ie, ≤5 minutes, 6–30 minutes, 31–60 minutes, >60 minutes) and cigarettes smoked per day (continuous), both entered on app download, once per participant.

#### Time-Varying Variables

When self-initiating a new craving feature entry, participants were asked to indicate their craving severity, how they are feeling, what they are doing, and who they are with. Although each craving entry is time-stamped, it is possible for the craving to have occurred some time ago (ie, retrospective reporting). Craving feature entries where the time difference between when the entry was made and the date/time the entry referred to was >24 hours were excluded to minimize reporting bias. The craving feature asked participants to indicate when the craving occurred.

Time-varying explanatory variables grouped under “Feeling states” included craving severity (as indicated on a 11-point Likert scale; 0-10), annoyance (no vs. yes), anxiety (no vs. yes), boredom (no vs. yes), sadness (no vs. yes), happiness (no vs. yes), hunger (no vs. yes), and loneliness (no vs. yes).

Time-varying explanatory variables grouped under “Activity” (no vs. yes) included whether they are at a bar or event, chatting, drinking coffee or tea, drinking alcohol, driving, going to bed, just eaten, just had sex, reading, relaxing, taking a break, thinking, waking up, and working.

Time-varying explanatory variables grouped under “Social context” (no vs. yes) included whether they are alone, with colleagues, with family, with friends, with their partner, and with strangers.

Time-varying explanatory variables grouped under “Temporal” included time of day (morning, midday, evening, and night), day of the week (Monday to Sunday), a derived cumulative time variable (in days) from the quit date, a variable capturing the time (in minutes) since users’ previous craving feature entry, and a variable capturing whether the previous craving feature entry was a lapse (no vs. yes).

The time-varying explanatory variables captured feeling states, social context, etc., when the craving was experienced rather than when the entry was self-initiated (when these differed).

### Data Analysis

The analyses were conducted in the R statistical programming language (v.4.1.1) with the *tidymodels* framework of packages,^27^ setting the engine to the relevant algorithm type (eg, “ranger” for Random Forest (RF) or “glmnet” for Penalized Logistic Regression) and the mode to “classification.” Four different types of supervised machine learning algorithms were trained and tested (ie, RF, Support Vector Machine (SVM), Penalized Logistic Regression, and Extreme Gradient Boosting), selected based on their relatively low computational demands (as the algorithm will ultimately be implemented within a smartphone app or similar), the availability of off-the-shelf R packages, and their relatively good interpretability compared with approaches such as deep learning (see the Supplementary Materials for an overview of the algorithms). As each algorithm uses different equations (with different numbers of terms) to estimate the model parameters, we aimed to compare their performance. Because of the nonuniform frequency of craving feature entries within and across participants, same-time (as opposed to lagged) predictor-outcome relationships were modeled.

#### Algorithm Training and Testing

Algorithm training and testing were performed through k-fold cross-validation,^28^ with *k* set to 10. For each iteration (or fold), algorithms were trained on 80% and tested on the remaining 20% of the data, with the exception of the hybrid group- and individual-level algorithms (see Supplementary Materials for additional methodological detail and Supplementary Figure S2 for an illustration of the train and test splits across the different objectives). The aim of the machine learning process is to minimize the out-of-sample error (ie, how accurately an algorithm can classify outcome values in a previously unseen sample). This is typically done by splitting the dataset (D) into a train set (D_train_) of size N-K and a test set (D_test_) of size K, with the latter used to estimate the out-of-sample error (as it was not used during the learning phase). If K is too small, the out-of-sample error will be wide; and if K is too large, the in-sample error (in the training set) will be wide. Hence, the two types of error need to be balanced, and a widely used rule of thumb is to set K = N/5—that is, setting aside 20% of the dataset for testing.^29^

Predicted and observed outcomes were first compared to estimate algorithm accuracy (ie, the proportion of true positives and true negatives), sensitivity (ie, the true positive rate), and specificity (ie, the true negative rate). Next, algorithm performance was evaluated by calculating an area under the receiver operating characteristic curve (AUC) estimate and an accompanying 95% confidence interval (CI) using the *pROC* package.^30^ The AUC captures the trade-off between sensitivity and specificity. AUC estimates with CIs that include 0.50 (ie, chance performance for a binary outcome) were considered unacceptable. For the group-level algorithms, we focused on the AUC as it provides an effective way to summarize the overall algorithm performance (ie, a single measure that takes both sensitivity and specificity into account, which precludes the need to present these separately). For the individual-level algorithms, however, estimates were compared with prespecified thresholds for acceptable accuracy (0.70), sensitivity (0.70), and specificity (0.50).^24^ It is more costly for a future JITAI to miss a true positive (lapse) than a true negative (non-lapse) because the former may set the individual on a trajectory towards full relapse, and with support provided in lower risk situations unlikely to be harmful to individuals, which explains our lower specificity threshold (0.50). We, therefore, considered it important to present these performance metrics separately for the individual-level algorithms. We also present the AUC to enable direct comparison with the group-level algorithms. See Supplementary Materials for additional details pertaining to each of the four study objectives, including sensitivity analyses conducted.

## Results

**Figure 1.**
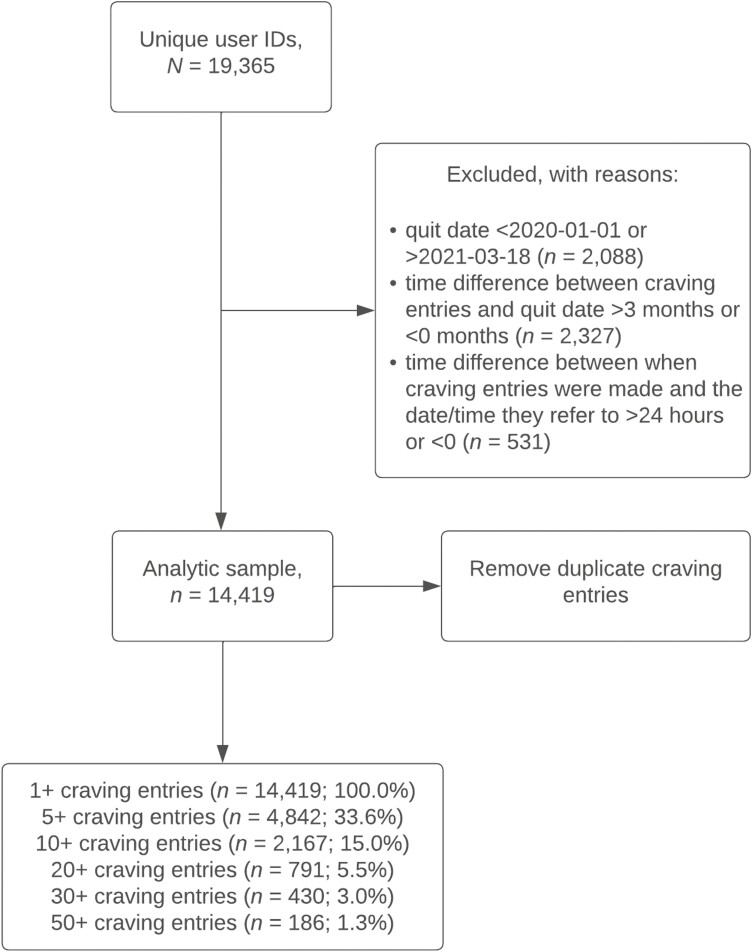
Participant flow chart.

A total of 19 365 participants registered to use the “pro” version of the Smoke Free app within the study period, with 14 419 (74.5%) participants eligible for inclusion (see Figure 1). Of these participants, 791 (5.5%) had made ≥20 craving entries within 3 months from the date of app registration and were included in the analyses, as a sufficient number of craving entries per participant was needed for the individual-level algorithms to run (see Table 1). To examine algorithm robustness, sensitivity analyses were conducted with a different cutoff (ie, participants with ≥10 craving entries).

**Table 1.** Participant Characteristics

	Total sample (*N* = 14 419)	Analytic sample with 20+ craving entries (*N* = 791)	Sensitivity sample with 10+ craving entries (*N* = 2167)
Cigarettes per day, mean (SD)	15.6 (*8.4*)	17.9 (9.0)	17.0 (8.5)
Time to first cigarette, *N* (%)			
≤5 min	3867 (26.8%)	246 (31.1%)	650 (30.0%)
6–30 min	5384 (37.3%)	307 (38.8%)	808 (37.3%)
31–60 min	2804 (19.4%)	148 (18.7%)	441 (20.4%)
>60 min	2364 (16.4%)	90 (11.4%)	268 (12.4%)
Craving entries, median (range)	3 (1, 686)	31 (20, 686)	16 (10, 686)
>5 recorded lapses, *N* (%)	127 (0.9%)	52 (6.6%)	103 (4.8%)
>5 recorded non-lapses, *N* (%)	3682 (25.5%)	779 (98.5%)	2116 (97.6%)
>5 recorded lapses and >5 recorded non-lapses, *N* (%)	54 (0.4%)	40* (5.1%)	54 (2.5%)

*Although 40 participants had sufficient data for the individual-level algorithms, performance metrics could still not be computed for 1 participant, resulting in a total *n* = 39 participants.

In the analytic sample (*n* = 791), participants provided a total of 37 002 craving entries with the median number of entries per participant being 31 (range: 20–686). The proportion of recorded lapse (vs. non-lapse) events across all craving entries were 7.6% (2816/37 002). The proportion of lapses (vs. non-lapses) varied widely across users, with a median of 0% lapses (range: 0%–100%; see Supplementary Materials, Figure S3).

### Objective 1 - Identifying a Best-Performing Group-Level Algorithm

**Figure 2.**
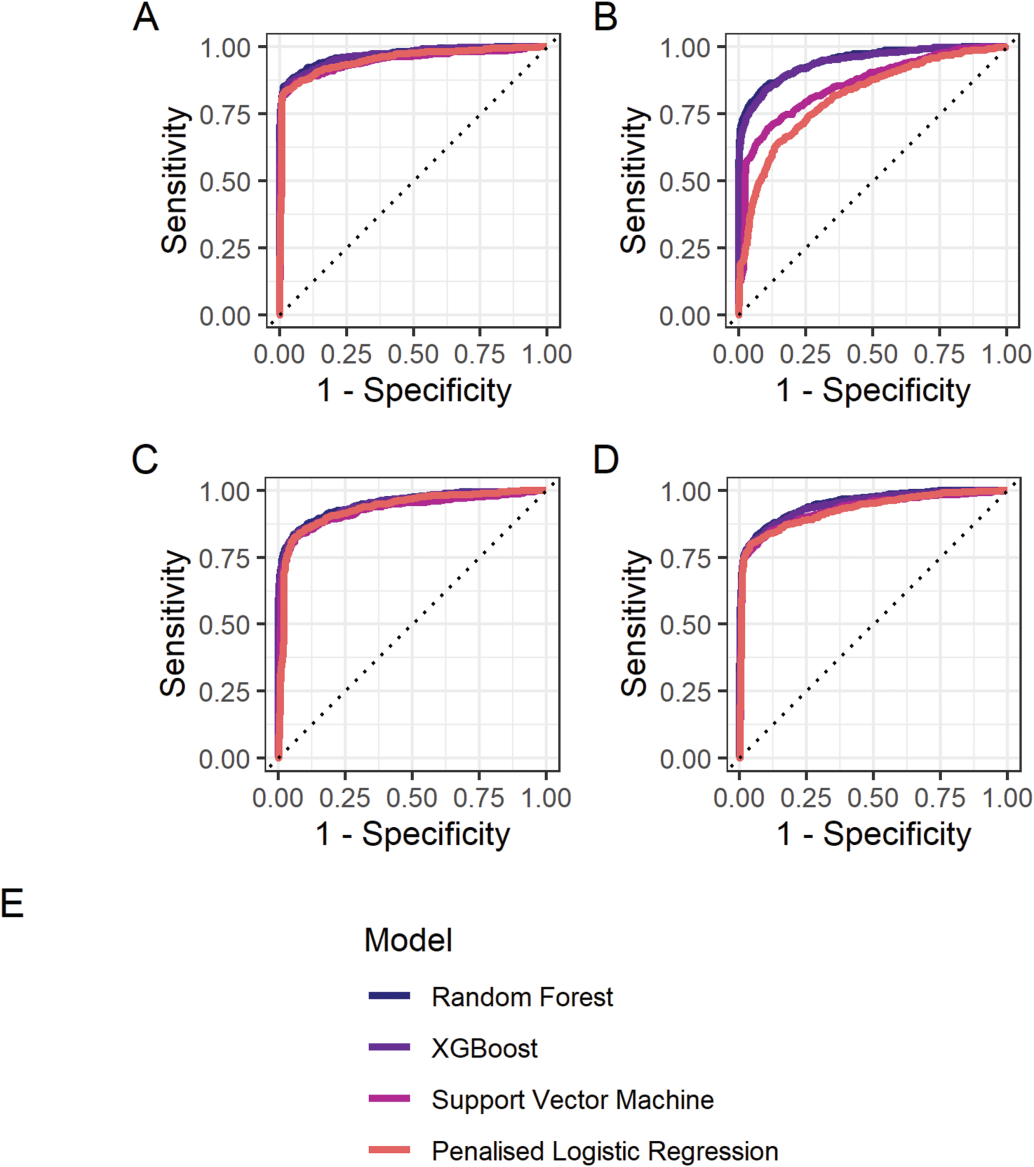
Panel (A) Plot of the area under the receiver operating characteristic curve (AUC) estimate for each of the group-level algorithms; panel (B) sensitivity analysis excluding the “prior event lapse” predictor variable; panel (C) sensitivity analysis excluding participants with 0% lapses; panel (D) sensitivity analysis using a different cutoff for inclusion in the analytic sample (ie, ≥10 craving entries).

The best-performing group-level algorithm was a RF algorithm (AUC = 0.969, 95% CI = 0.961 to 0.978; see Figure 2, panel A). This was closely followed by an Extreme Gradient Boosting (XGBoost) algorithm, with an AUC of 0.966 (95% CI = 0.958 to 0.975), a Penalized Logistic Regression algorithm (AUC = 0.952, 95% CI = 0.940 to 0.963), and a SVM algorithm (AUC = 0.947, 95% CI = 0.934 to 0.960). The parameter values and the variable importance for the best-performing group-level algorithms selected after tuning are presented in Supplementary Materials, Table S1, and Figure S4, respectively.

In a series of sensitivity analyses, algorithm performance remained largely robust for the best-performing RF (AUC = 0.947, 95% CI = 0.936 to 0.958) and XGBoost (AUC = 0.943, 95% CI = 0.931 to 0.954) algorithms when excluding the “prior event lapse” predictor variable (Figure 2, panel B). However, performance somewhat deteriorated for the SVM (AUC = 0.858, 95% CI = 0.840 to 0.876) and Penalized Logistic Regression (AUC = 0.815, 95% CI = 0.795 to 0.835) algorithms. When excluding participants with 0% lapses, performance for the RF (AUC = 0.950, 95% CI = 0.939 to 0.962), SVM (AUC = 0.928, 95% CI = 0.913 to 0.943), Penalized Logistic Regression (AUC = 0.934, 95% CI = 0.921 to 0.947), and XGBoost (AUC = 0.948, 95% CI = 0.936 to 0.959) algorithms remained largely robust (Figure 2, panel C). When using a different cutoff for inclusion in the analytic sample (ie, ≥10 craving entries), algorithm performance remained largely robust for the best-performing RF (AUC = 0.952, 95% CI = 0.943 to 0.960), SVM (AUC = 0.929, 95% CI = 0.917 to 0.941), Penalized Logistic Regression (AUC = 0.928, 95% CI = 0.916 to 0.940), and XGBoost (AUC = 0.947, 95% CI = 0.938 to 0.957; Figure 2, panel D).

**Figure 3.**
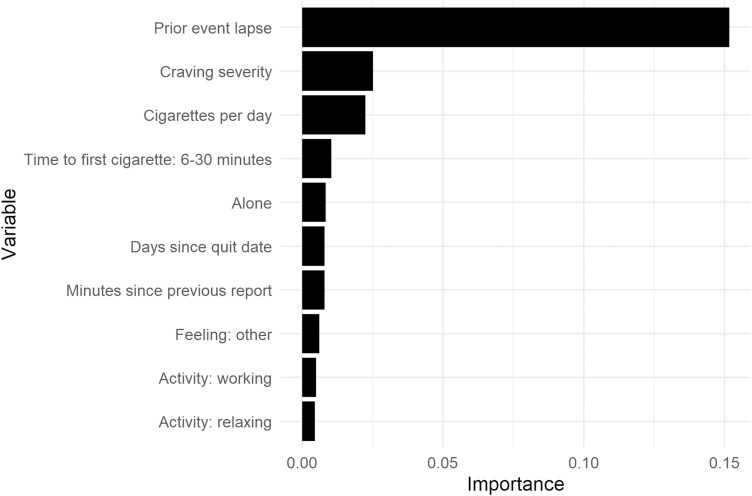
Variable importance plot for the best-performing group-level Random Forest algorithm. The variable importance score does not indicate the direction of the relationship between the predictor and outcome variable.

The most influential predictor variables for the original, best-performing group-level RF algorithm included whether or not the immediately preceding event was a lapse (time varying), craving severity (time varying), cigarettes smoked per day (time invariant), time to first cigarette (time invariant), and whether the person was alone (time varying; see Figure 3).

### Objective 2 - Performance of the Best-Performing Group-Level Algorithm for Out-of-Sample Individuals

After removing participants with 0% or 100% lapses, algorithm performance could be computed for *n* = 201 (25.4%) participants. The median AUC was high at 0.839; however, this metric varied widely across participants (range: 0.482–1.000).

### Objective 3 - Identifying Best-Performing Individual-Level Algorithms

**Figure 4.**
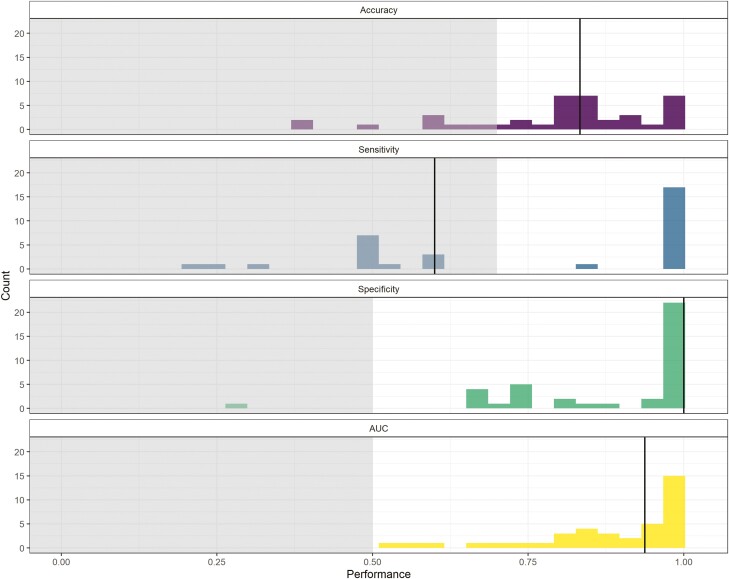
Frequency distributions of the performance metrics of interest (ie, accuracy, sensitivity, specificity, AUC) for the best-performing individual-level algorithms (*n* = 39). The shaded gray areas represent the prespecified thresholds for acceptable accuracy (0.70), sensitivity (0.70), specificity (0.50), and AUC (0.50). The solid vertical lines represent the median.

After removing participants with insufficient data, algorithm performance metrics could be computed for *n* = 39 (4.9%) participants. Based on the AUC, the best-performing individual-level algorithms were of the type RF (43.6% of participants; 17/39), Penalized Logistic Regression (30.8% of participants; 12/39), SVM (17.9% of participants; 7/39), and XGBoost (7.7% of participants; 3/39). Figure 4 illustrates the frequency distribution of the performance metrics of interest for participants’ best-performing algorithms. The median AUC for participants’ best-performing algorithms was 0.938 (range: 0.518 to 1.000).

In a sensitivity analysis examining the number of participants for whom the individual-level algorithm provided a benefit over the group-level algorithm, the individual-level algorithm was superior for the majority of participants (31/39; 79.5%). For the participants for whom the group-level algorithm was superior (8/39; 20.5%), a substantial benefit was observed only for a small minority of participants (see Supplementary Materials, Figure S5).

Next, we examined the proportion of participants with each of the predictor variables in their top 10 from their best-performing individual-level algorithm (*n* = 39; see Supplementary Materials, Figure S6). For example, craving severity and whether the prior event was a lapse were included in 50% of participants’ top 10 lists.

### Objective 4 - Performance of a Hybrid (Group- and Individual-Level) Algorithm

When repeating the analyses conducted to address Objective 2 but with 20% of the individual’s data included in the training set (*n* = 184), the median AUC was 0.825 (range: 0.375 to 1.000). The hybrid algorithm was superior to the group-level algorithm for 51.6% (95/184) of participants.

In a sensitivity analysis with 40% of the individual’s data included in the training set (*n* = 158), the median AUC remained largely robust at 0.824 (range: 0.392 to 1.000). This hybrid algorithm provided a benefit over the group-level algorithm for a slightly greater proportion (92/158; 58.2%) of participants.

## Discussion

This study aimed to train and test a series of group- and individual-level supervised machine learning algorithms to distinguish lapse from non-lapse events in smokers attempting to quit with the support of a popular smartphone app. A RF algorithm—which drew most heavily on a combination of time-varying (eg, whether the immediately preceding event was a lapse, craving severity, whether the person was alone) and time-invariant (eg, cigarettes smoked per day, time to first cigarette after waking up) predictor variables—classified out-of-sample observations with high levels of accuracy, sensitivity, and specificity. However, when the best-performing group-level algorithm was used to classify lapses for unseen individuals (which would ultimately be the aim of a technology-mediated JITAI), performance was variable. Individual-level algorithms trained and tested on each individual’s data led to improved performance but could only be constructed for a minority of participants with a sufficient number of recorded lapse and non-lapse events. Finally, a hybrid algorithm trained on the group plus a proportion of each individual’s data (20%–40%) led to somewhat improved performance compared with the group—but not individual-level algorithm and could be constructed for a much larger proportion of participants (ie, 23.3% vs. 4.9%). It is plausible that the hybrid algorithms need additional individual data to provide a benefit over the group-level algorithm.

Our results add to those from a recent body of work in which supervised machine-learning algorithms have been developed to classify or predict smoking-related events.^12–14,17^ In addition, we drew on a recent approach taken to train and test individual-level algorithms to predict dietary lapses,^23^ alcohol consumption,^31^ and loneliness and procrastination,^32^ with a view to informing the development of a technology-mediated JITAI. Although useful, individual-level algorithms could only be developed for a minority of participants in the present study. This may be interpreted to suggest that such an individual-level approach may not be feasible with routinely collected smoking cessation app data. In addition, as we, through a systematic approach, settled on including smokers who had reported >5 lapses and >5 non-lapses to train and test the individual-level algorithms, it is important to note that this subsample differed from the total sample with regards to key smoking characteristics (and possibly also sociodemographic characteristics). However, there was relatively little encouragement or incentive for participants to engage with the craving feature regularly and there was no indication within the app that providing such data could help to develop better support. Adding these elements into an app could improve engagement and may extend the proportion of participants for whom this individual-level approach could be feasible (discussed further below).

### Strengths and Limitations

This study was strengthened by drawing on routinely collected craving feature data from a popular smoking cessation app, with the data structure representing real-world conditions. Specifically, the data structure provides a realistic estimate of how much data to expect and what the data will look like in an unprompted study design. The study was also strengthened by the involvement of an interdisciplinary team of researchers working across the diverse fields of tobacco control, behavioral science, data science, and machine learning.

There were also important limitations. First, the occurrence of lapses was self-reported (rather than biochemically verified or relying on passive sensing) and although the unprompted and non-incentivized study design represented real-world conditions, it likely influenced the data quality. For example, app users may have been less likely to self-initiate a new craving feature entry when they had lapsed. The distribution of the proportion of recorded lapses (vs. non-lapses) lends support to this: most participants in the analytic sample reported 0% lapses. It remains an empirical question as to whether a similar pattern of lapses (vs. non-lapses) would be observed if using a prompted study design. However, in a recent study using EMAs, 56.5% of the sample recorded at least one lapse in the first week of the quit attempt,^8^ which suggests that the large proportion of participants with 0% lapses in the present study is likely reflective of self-selection bias, which may have materialized in two different ways. First, by setting the cutoff for inclusion to ≥20 diary entries, we may have excluded users who lapsed early in the quit attempt and disengaged from the app, thus retaining only the most highly engaged users (and also successful quitters). The digital health literature shows a pattern of “reverse causality,” with those continuing to engage with digital health tools being more likely to remain abstinent from smoking.^33,34^ Alternatively, the observed pattern may reflect a bias with regard to how smokers engaged with the app: participants may have been prone to use the craving feature to record situations that did not result in a lapse. As our sample included participants with 100% lapses, this lends further support to the argument that the distribution of lapses in the present study is likely more a reflection of how participants self-selected to engage with the app rather than the actual course of events. In a study using a prompted design, one would not expect any users with 100% lapse entries. We, therefore, recommend repeating the analyses using data from a prompted study design (eg, using both signal- and event-contingent EMAs), with results triangulated with those from the present study to arrive at a better understanding of the trajectory of lapses.

Second, also due to the unprompted study design, the frequency of and temporal distance between craving feature entries were not uniform across participants. Craving feature entries were typically made several days rather than hours apart. We were therefore unable to lag measurements, which is typically done in EMA studies (eg, cravings measured at t_1_ are typically used to predict lapses at t_2_). Therefore, the present study was limited by modeling same-time (or “contemporaneous”) predictor-outcome associations, with participants potentially recording a lapse and inferring (at the time of reporting) that they must therefore have experienced a strong craving or felt stressed. In addition, the nonuniform frequency of and temporal distance between craving feature entries also means that it took participants varying amounts of time to reach the minimum of 20 craving feature entries (and, by extension, the >5 lapses and >5 non-lapse reports required for the individual-level algorithms), which may have influenced both algorithm performance and variable importance scores. Related to this, we observed that different predictor variables were estimated as important for the different group-level algorithm types (eg, the variable “prior event lapse” was estimated as key in three of four group-level models, but there was considerable variability in the other predictors estimated as important). This may be interpreted to suggest that care needs to be taken by researchers prior to designing JITAIs that rely on estimates from the variable feature importance function or similar functions (eg, a JITAI designed to deliver a specific type of intervention/message based on the variables estimated as being most important for predicting lapse incidence), irrespective of whether the algorithm is operating at the group- or individual-level. In addition, a series of sensitivity and stability analyses (eg, partial dependence plots) are recommended to ensure that predictor variables remain robust across different mathematical formalisms (ie, algorithm types), as results can be sensitive to aspects such as collinearity of the predictor variables.^35^ Such robustness analyses, in addition to external validation, are needed prior to providing additional interpretation as to which predictor variables appear most important and why and explains why we remain cautious here as to the interpretation of the results from the variable importance function.

Third, there is evidence to suggest that the likelihood of self-initiating craving feature entries during/after particular events (eg, drinking alcohol, lapsing, and socializing) likely varies,^36^ which may have affected the apparent importance of particular variables in the classification of lapses.

Fourth, the supervised machine learning algorithms tested in the present study assume the independence of observations. Although the approach taken was deemed appropriate given the aims of the present study, future research would benefit from exploring more advanced methods (eg, multilevel or recurrent neural network algorithms) that better account for nested observations within individuals over time.

Finally, as the dataset was imbalanced, random up- or down-sampling with replacement was used prior to algorithm training and testing. However, it has been found that imbalance correction through random up- or down-sampling can sometimes lead to poorly calibrated models, with the probability of belonging to the minority class being overestimated.^37^

### Implications for Research and Practice

Pending data on the distribution of lapses (vs. non-lapses) at the within-person level in a prompted study design, a different outcome variable with greater within-person variability and frequency (eg, craving severity) may be needed. As a few early lapses typically lead to full relapse,^38^ there may be insufficient variation in the outcome for individual-level modeling. However, the individual-level algorithms performed better than the group-level algorithms for the minority of participants with a sufficient number of recorded lapse and non-lapse events. Thus, we would argue that—drawing also on data from several other studies highlighting the idiosyncratic nature of lapse risk in smokers attempting to stop, with different factors important for different individuals^7–11^—there is merit in further exploring the potential of the individual-level, or hybrid group- and individual-level, algorithms. Future work would also benefit from external validation of the algorithms trained and tested here in a different sample.

As we used routinely collected data from a popular smoking cessation app, we did not incorporate several variables that are known to be strongly associated with lapse risks, such as cigarette availability or self-efficacy.^7,10^ Apart from the craving severity variable, the items used to capture the psychological and contextual information were limited to binary response options. Future work would benefit from incorporating a wider range of theoretically and empirically informed predictor variables, using items with a wider range of response options to increase power to detect potentially subtle associations.

Finally, the frequent self-reports required for accurate individual-level classification may not be feasible under real-world conditions and some may argue that JITAIs are limited insofar as they require frequent active user input (eg, EMAs) in order to function (eg, to estimate the need for support and what type of support would be most effective). Similar to recent work conducted in the smoking and dietary lapse fields,^39,40^ exploring the predictive power of passively collected sensor data (eg, step count, GPS, heart rate variability) is an important avenue for future research.

## Conclusion

Using unprompted app data appeared feasible for constructing a high-performing group-level lapse classification algorithm but its performance was variable when applied to unseen individuals. Algorithms trained on each individual’s dataset, in addition to hybrid algorithms trained on the group plus a proportion of each individual’s data, had improved performance but could only be constructed for a minority of participants.

## Supplementary Material

A Contributorship Form detailing each author’s specific involvement with this content, as well as any supplementary data, are available online at https://academic.oup.com/ntr.

ntad051_suppl_Supplementary_Materials

## Data Availability

The data and R code underpinning the analyses are available on GitHub (https://github.com/OlgaPerski/lapses_smokefree_ml).
