# Supplementary material for: Classification of Lapses in Smokers Attempting to Stop: A Supervised Machine Learning Approach Using Data From a Popular Smoking Cessation Smartphone App
Source: Nicotine Tob Res. 2023 Mar 27;25(7):1330–9. doi: 10.1093/ntr/ntad051 (PMC10256890; doi:10.1093/ntr/ntad051)
Supplement: ntad051_suppl_Supplementary_Materials [file ntad051_suppl_supplementary_materials.docx]

**Supplementary Materials**

***Supervised machine learning algorithms***

*Random Forest*

The Random Forest (RF) algorithm uses ‘ensemble modelling’ (i.e., a combination of many decision trees, or a ‘forest’) to output predicted class labels by relying on the majority vote, which prevents overfitting^1^. Each tree is represented by a series of nodes (i.e., predictors), at which data are split. The order of nodes to split on is determined by their relative ability to reduce entropy (or uncertainty). Decision trees are built by taking a random subset of the training set, randomly selecting a subset of available predictors at each node and selecting the predictor that provides the greatest reduction in entropy. This process is repeated until the tree has grown to a pre-defined depth or no additional split can reduce entropy – also known as ‘recursive partitioning’. The process of growing a decision tree is then repeated multiple times to generate a forest. The number of trees to grow is typically pre-specified by the researcher, with little additional benefit typically observed after growing 500 trees^2^. The final predicted class label is produced through aggregating predictions from the individual decision trees, with the most frequent prediction selected (i.e., the majority vote). With regards to model-specific hyperparameters in RFs, researchers typically tune the subset of predictors to try at each node and the minimum number of observations in a node required for further splitting.

*Support Vector Machine*

The Support Vector Machine (SVM) algorithm aims to separate the data points through identifying a hyperplane in N-dimensional space, with a view to maximising the space (or ‘margin’) between the hyperplane and data points^3^. The SVM is a linear classifier but can be extended to a non-linear one through the application of a ‘kernel function’ (e.g., linear, polynomial, radial basis function, sigmoid), which projects data from a low- to a high-dimensional space (e.g., from two to three dimensions). We used the general-purpose (and the most commonly applied) radial basis kernel function^4^, which has two key parameters to tune – cost and sigma. The former corresponds to the cost of making a prediction within or on the wrong side of the margin and the latter corresponds to the precision parameter for the radial basis function.

*Penalised Logistic Regression*

Penalised Logistic Regression algorithms use ‘regularisation’ to penalise models depending on the number of predictors by shrinking coefficients that do not contribute substantially towards zero. We used an elastic net model, which combines features from both ridge and lasso regression in that they shrink some coefficients towards zero (similar to ridge regression) and set some to exactly zero (similar to lasso regression). Researchers can tune the amount of shrinkage (often referred to as the ‘penalty’) and the ‘mixing parameter’ (with 1 = lasso regression, 0 = ridge regression, and values between 0 and 1 = elastic net regression).

*Extreme Gradient Boosting*

The Extreme Gradient Boosting (XGBoost) algorithm has evolved from the RF, with two notable exceptions: while RFs build each decision tree independently, XGBoost algorithms learn from previous shortcomings and improve performance for each consecutive tree built and while RFs combine predicted class labels at the end of the process, XGBoost algorithms sequentially combine predictions from individual decision trees^5^. In addition, similar to Penalised Logistic Regression, XGBoost algorithms use regularisation to prevent overfitting. Model-specific hyperparameters to tune, in addition to those described in relation to the RF algorithm above, include the maximum tree depth (i.e., the maximum number of node splits), the learn rate (i.e., the rate at which the algorithm adapts from iteration to iteration), the loss reduction (i.e., the reduction in the loss function required for further node splits), and the sample size (i.e., the proportion of data exposed to the fitting process).

***Additional details pertaining to each objective, with sensitivity analyses***

*Objective 1 - Identifying a best-performing group-level algorithm*

First, as the dataset was imbalanced with a greater number of non-lapse than lapse events, random down-sampling was used to balance the training set. Down-sampling (as opposed to up-sampling) was selected due to the size of the dataset – i.e., up-sampling substantially increased algorithm run times and was therefore not considered feasible. Next, an optimal group-level algorithm (e.g., Random Forest, Support Vector Machine) was identified. Algorithm-specific hyperparameters were tuned to minimise the generalisation error and optimise algorithm performance^6^. For algorithms with a large number of hyperparameters (i.e., Extreme Gradient Boosting), space-filling was used to construct manageable hyperparameter search spaces using the Latin hypercube design, which constructs parameter grids that attempt to cover the entire parameter space but without testing every possible configuration^7^. For the best-performing group-level algorithm, the *vip* package^8^ was used to estimate the permutation-based, model-agnostic feature importance, as a future technology-mediated JITAI would use this information to tailor real-time support to the most influential predictor variables.

*Sensitivity analyses*

After examining the data, we noticed three patterns that could impact algorithm performance. We therefore conducted a series of unplanned sensitivity analyses to directly address these conceptual concerns. We first examined the impact on algorithm performance of excluding the predictor variable capturing whether the immediately preceding event was a lapse. This was to test whether the small proportion of lapsers in the dataset was the primary driver of algorithm performance. Second, likely due to the unprompted study design, most participants either did not have a single lapse or self-selected to only engage with the app to report cravings/non-lapse events (referred to henceforth as 0% lapses). We therefore examined the impact of excluding participants with 0% lapses on algorithm performance. Third, to test the robustness of the algorithm performance when using a different cut-off for inclusion in the analytic sample, we examined the impact of including participants with ≥10 (rather than ≥20) craving entries.

*Objective 2 - Performance of the best-fitting group-level algorithm for out-of-sample individuals*

Leave-one-out cross-validation was used to examine the performance of the best-performing group-level algorithm for ‘unseen’ individuals, who were each omitted from the training set and used for testing (i.e., the procedure was repeated for each participant in the dataset). To compute the algorithm performance metrics of interest, the training and testing data need to contain both lapse and non-lapse events. We therefore removed participants with 0% or 100% lapses.

*Objective 3 - Identifying best-performing individual-level algorithms*

Next, we took a systematic approach to identifying participants with data sufficient for training and testing the individual-level algorithms. As above, we first removed participants with 0% or 100% lapses. Next, we systematically varied the cut-off for the minimum number of lapses and non-lapses in each individual’s dataset to maximise the number of participants included in the analysis. When setting the cut-off to >5 recorded lapses and >5 recorded non-lapses, algorithm performance metrics could be computed for *n* = 39 participants.

Next, algorithms were separately trained and tested on each individual’s data. First, random up-sampling was used to balance the training sets. In contrast to the group-level algorithms, each individual’s dataset was relatively small, with short algorithm run times. Up-sampling was therefore considered feasible. Optimal individual-level algorithms were identified. Algorithm-specific hyperparameters were tuned to optimise performance.

We report the median AUC for participants’ best-performing algorithms in addition to the algorithms’ accuracy, sensitivity and specificity in comparison with the pre-specified thresholds. We also report the proportion of participants with the available predictor variables in their top 10, estimated with the *vip* function^9^.

*Sensitivity analysis*

We examined for how many participants the individual-level algorithm provided an improvement over the group-level algorithm, operationalised as the proportion of participants with an individual-level algorithm AUC that was equal to or greater than their group-level algorithm AUC.

*Objective 4 - Performance of a hybrid algorithm for individuals*

Next, the analyses conducted as part of Objective 2 were repeated, with 20% of each individual’s data included in the training sets. The remaining 80% of the individual’s data was used for testing. As above, we first removed participants with 0% or 100% lapses in addition to participants with an insufficient number of lapse or non-lapse events retained in the test set.

*Sensitivity analyses*

First, we examined for how many participants the hybrid algorithm provided a benefit over the group-level algorithm. Second, as a robustness check, we repeated the analyses with 40% of each individual’s data included in the training sets. The remaining 60% of the individual’s data was used for testing.

***References***

1. Breiman L. Random Forests. *Machine Learning*. 2001;45(1):5-32. doi:10.1023/A:1010933404324

2. Probst P, Boulesteix AL. To tune or not to tune the number of trees in random forest. *J Mach Learn Res*. 2017;18(1):6673-6690.

3. Cortes C, Vapnik V. Support-vector networks. *Mach Learn*. 1995;20(3):273-297. doi:10.1007/BF00994018

4. Patle A, Chouhan DS. SVM kernel functions for classification. In: *2013 International Conference on Advances in Technology and Engineering (ICATE)*. ; 2013:1-9. doi:10.1109/ICAdTE.2013.6524743

5. Chen T, Guestrin C. XGBoost: A Scalable Tree Boosting System. *Proceedings of the 22nd ACM SIGKDD International Conference on Knowledge Discovery and Data Mining*. Published online August 13, 2016:785-794. doi:10.1145/2939672.2939785

6. Probst P, Boulesteix AL, Bischl B. Tunability: Importance of Hyperparameters of Machine Learning Algorithms. *Journal of Machine Learning Research*. 2019;20(53):1-32.

7. Gramacy RB. *Surrogates*. CRC Press; 2020. Accessed January 7, 2022. https://bookdown.org/rbg/surrogates/

8. Greenwell BM, Boehmke BC, McCarthy AJ. A Simple and Effective Model-Based Variable Importance Measure. Published online May 12, 2018. Accessed January 6, 2022. http://arxiv.org/abs/1805.04755

9. Wickham H, Averick M, Bryan J, et al. Welcome to the Tidyverse. *JOSS*. 2019;4(43):1686. doi:10.21105/joss.01686


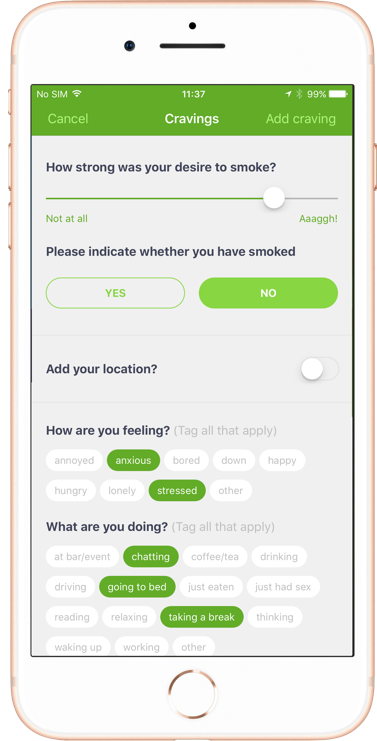


*Figure S1.* Screenshot of Smoke Free’s craving feature.


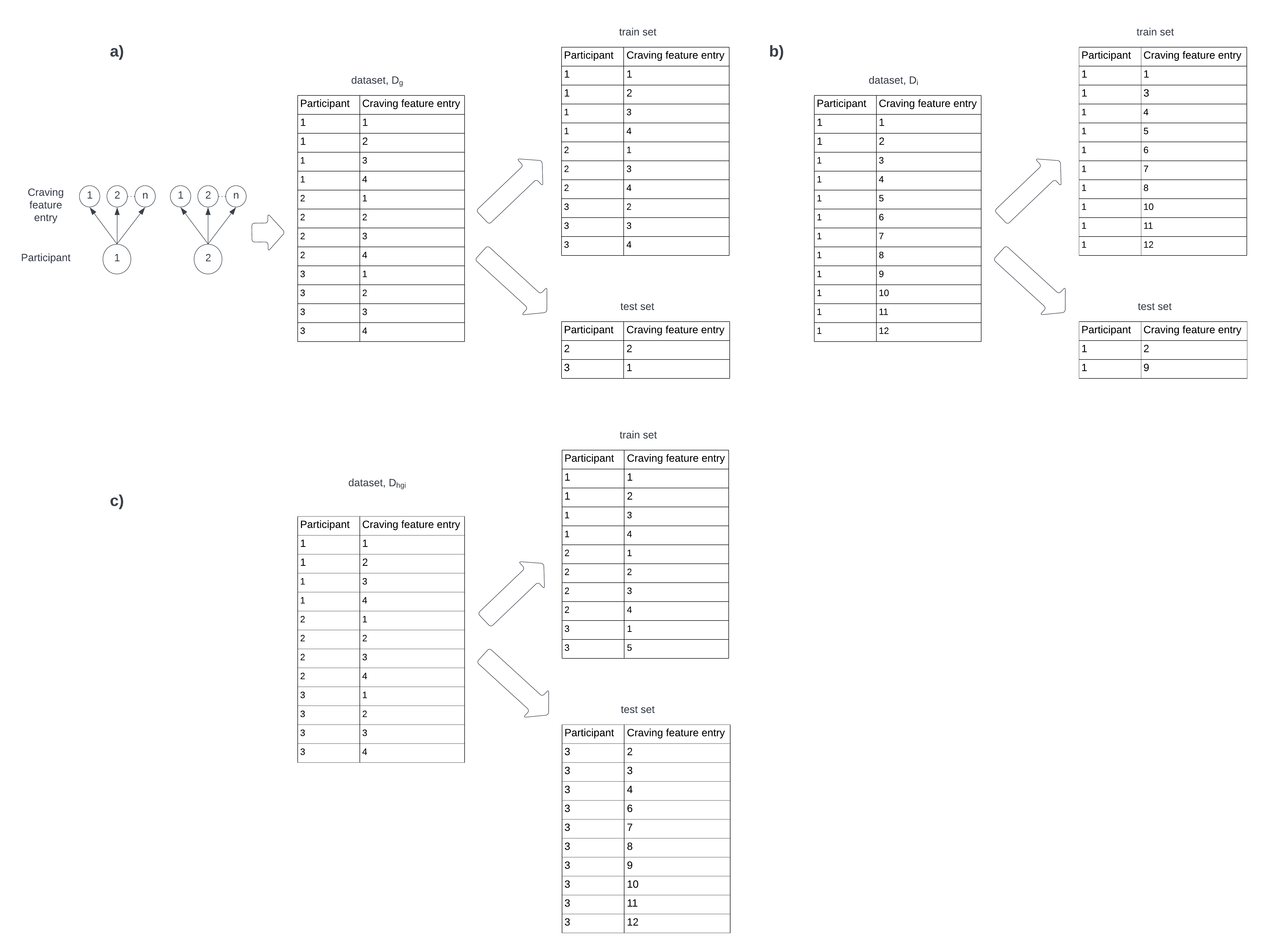


*Figure S2.* Panel **a)** The group-level dataset (D_g_) contained N_g_ = 37,002 observations, of which 20% of observations (n = 7,400) were randomly selected to be in the test set (K_g_). The train set contained 80% of the remaining observations (N_g_-K_g_; n = 29,602). Panel **b)** The individual-level datasets (D_i_) ranged from N_i_ = 20-686 observations, of which 20% of observations (n = 4-137) were randomly selected to be in the test sets (K_i_). The train sets contained 80% of the remaining observations (N_i_-K_i_; n = 16-549). Panel **c)** For the hybrid group- and individual-level algorithms, the datasets (D_hgi_) contained the group-level dataset, D_g_, minus the specific individual-level dataset at hand (looping through each participant in the dataset), D_i_, with 20% of the individual’s data randomly selected to be added back into the train set. The remaining 80% of each individual’s data was used as the test set (K_hgi_).


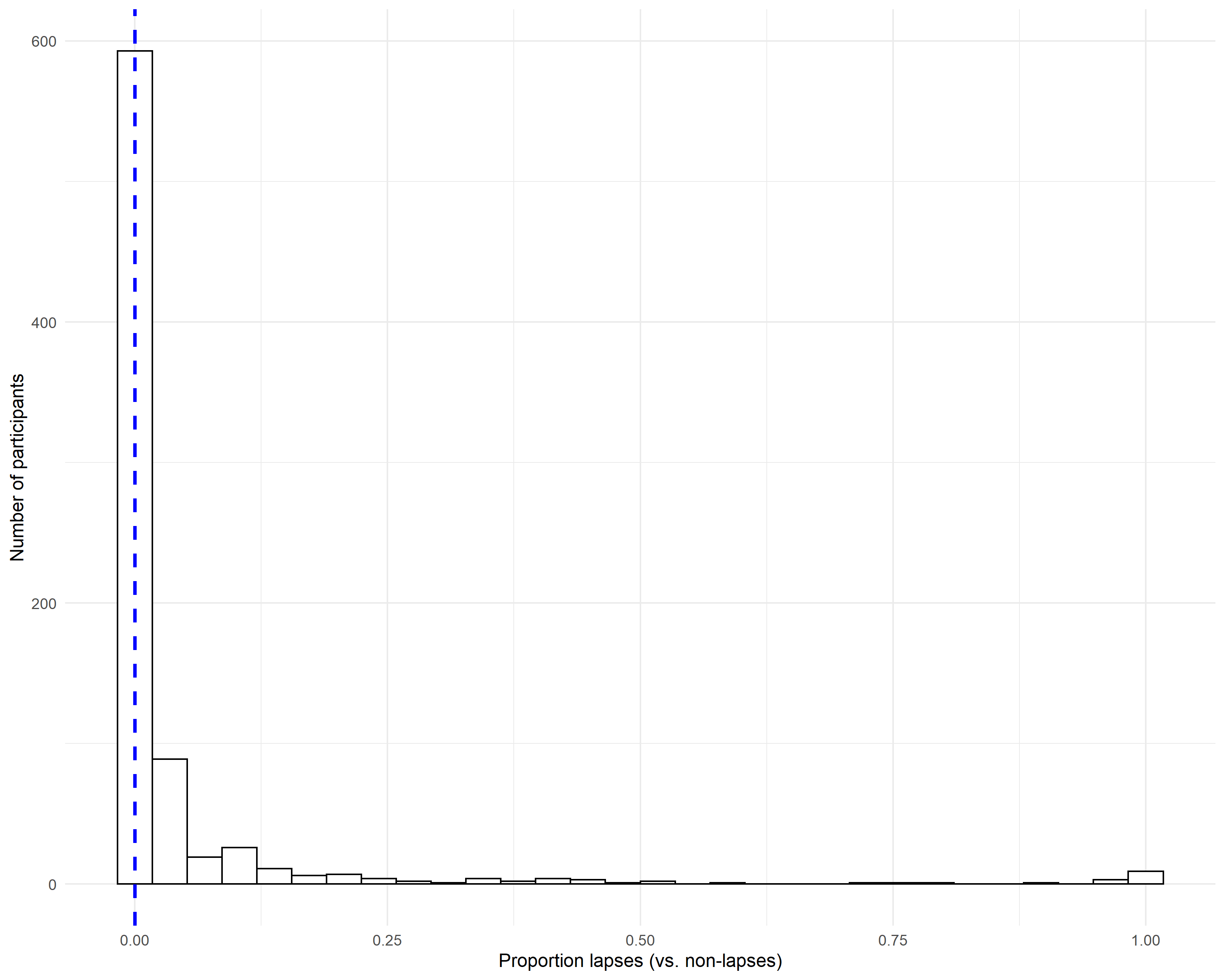


*Figure S3.* Frequency distribution of the proportion of recorded lapses (vs. non-lapses) for each user (*N* = 791).


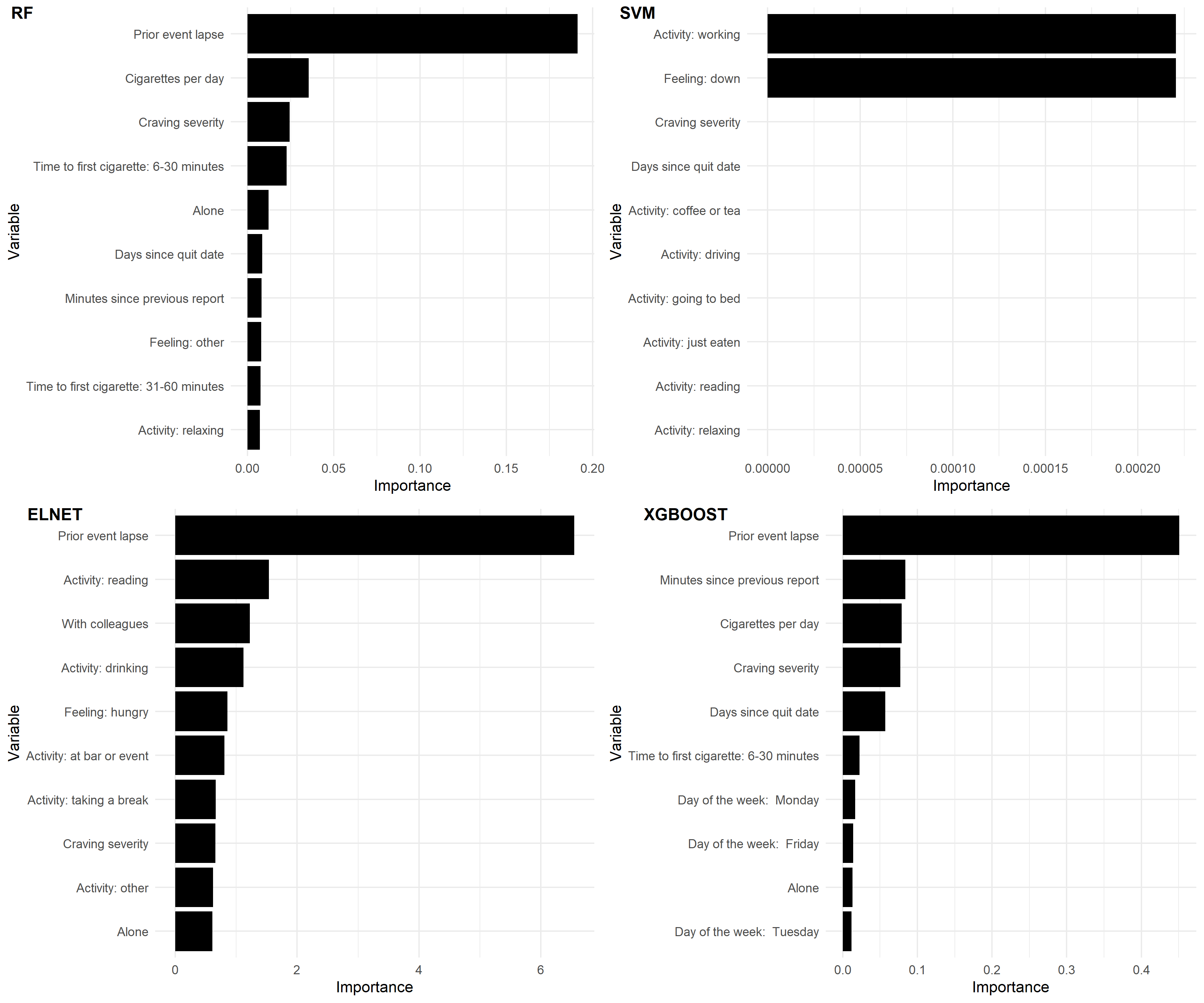


*Figure S4.* Variable feature importance for the best-performing group-level algorithms. RF = random forest; SVM = Support Vector Machine; ELNET = Penalised Logistic Regression; XGB = Extreme Gradient Boosting.


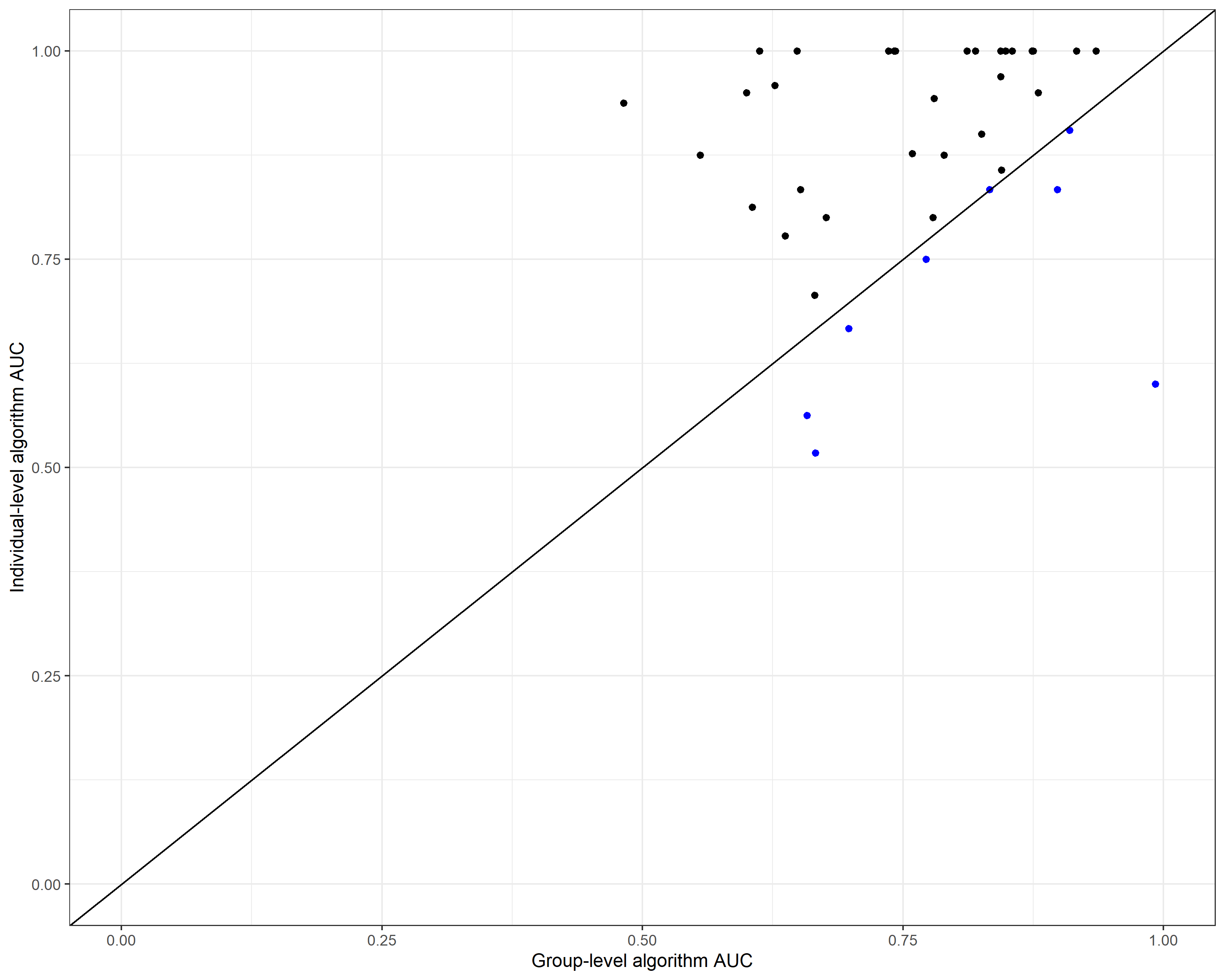


*Figure S5.* Comparison of the individual- and group-level algorithm performance for each individual (*n* = 39). Black dots indicate participants for whom the individual-level algorithm was superior to the group-level algorithm AUC. Blue dots indicate participants for whom the group-level algorithm AUC was superior to the individual-level algorithm AUC.


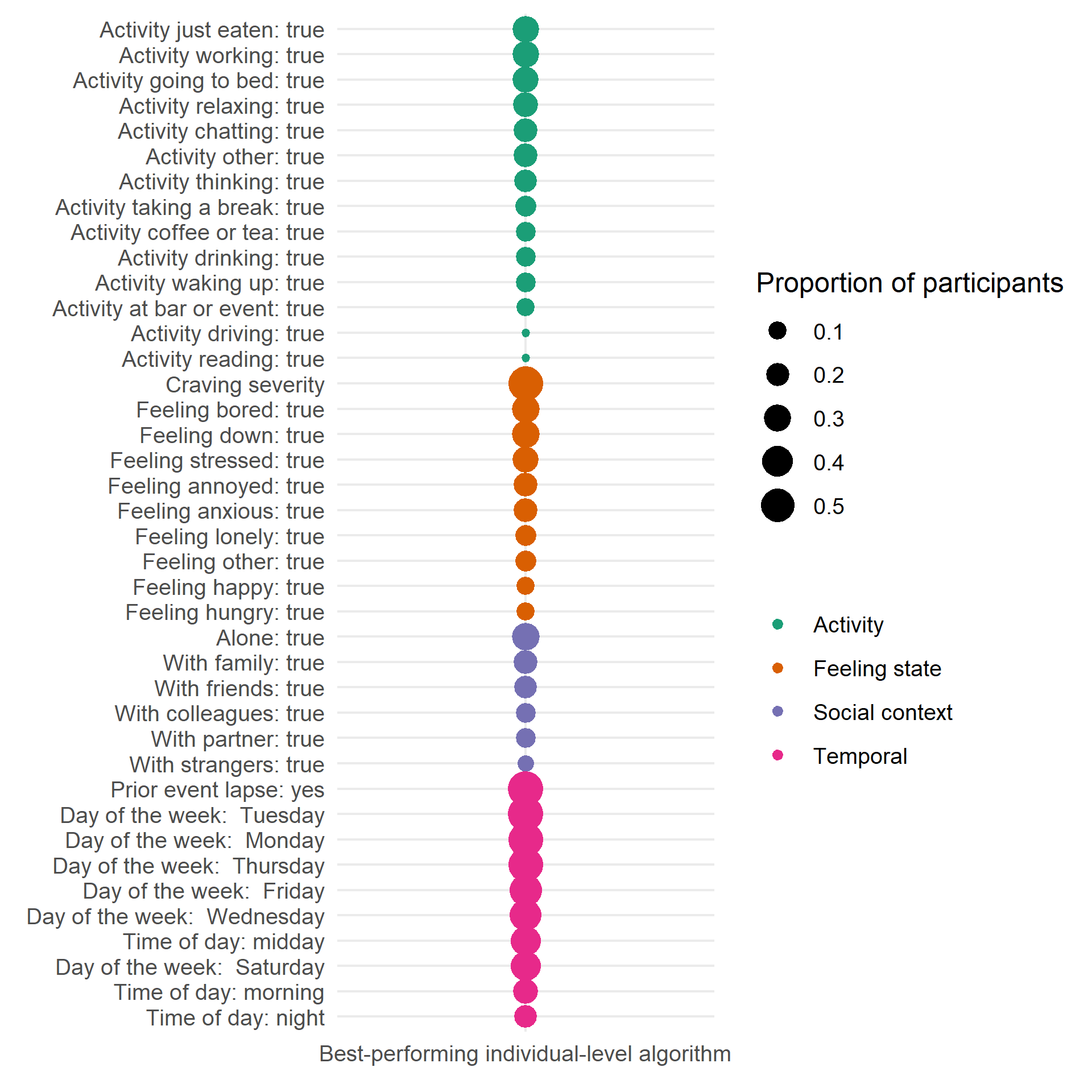


*Figure S6.* Proportion of participants with each of the predictor variables in their top 10 (*n* = 39). For clarity, predictor variables that were not included in a single participant’s top 10 are not displayed.

*Table S1.* Parameter values for the best-performing group-level algorithms.

|  | **mtry** | **trees** | **min_n** | **cost** | **tree_depth** | **learn_rate** | **loss_reduction** | **sample_size** | **rbf_sigma** | **penalty** | **mixture** |
| --- | --- | --- | --- | --- | --- | --- | --- | --- | --- | --- | --- |
| **RF** | 3 | 500 | 13 |  |  |  |  |  |  |  |  |
| **SVM** |  |  |  | 9.120 |  |  |  |  | 0.002 |  |  |
| **ELNET** |  |  |  |  |  |  |  |  |  | 0.009 | 0.677 |
| **XGB** | 15 | 1000 | 2 |  | 12 | 0.011 | 0.00002 | 0.423 |  |  |  |

*Note.* RF = random forest; SVM = Support Vector Machine; ELNET = Penalised Logistic Regression; XGB = Extreme Gradient Boosting.
